# Functions and mechanisms of eukaryotic RNA-guided programmed DNA elimination

**DOI:** 10.1042/BST20253006

**Published:** 2025-04-29

**Authors:** Bozhidar-Adrian Stefanov, Mariusz Nowacki

**Affiliations:** Institute of Cell Biology, University of Bern, Baltzerstrasse 4, Bern 3012, Switzerland

**Keywords:** ciliates, DNA elimination, genome editing, genome integrity, genome rearrangement

## Abstract

Many eukaryotic organisms, from ciliates to mammals, employ programmed DNA elimination during their postmeiotic reproduction. The process removes specific regions from the somatic DNA and has broad functions, including the irreversible silencing of genes, sex determination, and genome protection from transposable elements or integrating viruses. Multiple mechanisms have evolved that explain the sequence selectivity of the process. In some cases, the eliminated sequences lack centromeres and are flanked by conserved sequence motifs that are specifically recognized and cleaved by designated nucleases. Upon cleavage, all DNA fragments that lack centromeres are lost during the following mitosis. Alternatively, specific sequences can be destined for elimination by complementary small RNAs (sRNAs) as in some ciliates. These sRNAs enable a PIWI-mediated recruitment of chromatin remodelers, followed up by the precise positioning of a cleavage complex formed from a transposase like PiggyBac or Tc1. Here, we review the known molecular interplay of the cellular machinery that is involved in precise sRNA-guided DNA excision, and additionally, we highlight prominent knowledge gaps. We focus on the modes through which sRNAs enable the precise localization of the cleavage complex, and how the nuclease activity is controlled to prevent off-target cleavage. A mechanistic understanding of this process could enable the development of novel eukaryotic genome editing tools.

## Programmed DNA elimination

Maintaining genetic information accurately is essential for proper cellular functions. Perturbations impede the evolutionary fitness, through multimodal effects including cancer evolution [1,2], and cause resistance to cytotoxic immune responses [3], anti-cancer drugs [3], and development of metastasis [4]. Chromosomal error during embryonic development, such as DNA elimination due to stress and mitotic errors [5], or cytoplasmic DNA shedding [6] leading to aneuploidy, is a leading cause of pregnancy loss [6] or genetic disorders in the offspring [7]. Erroneous DNA elimination is thus a natural phenomenon related to genomic instability and causes a decrease in organismal fitness, since the eliminated sequences are random.

**Figure 1:**
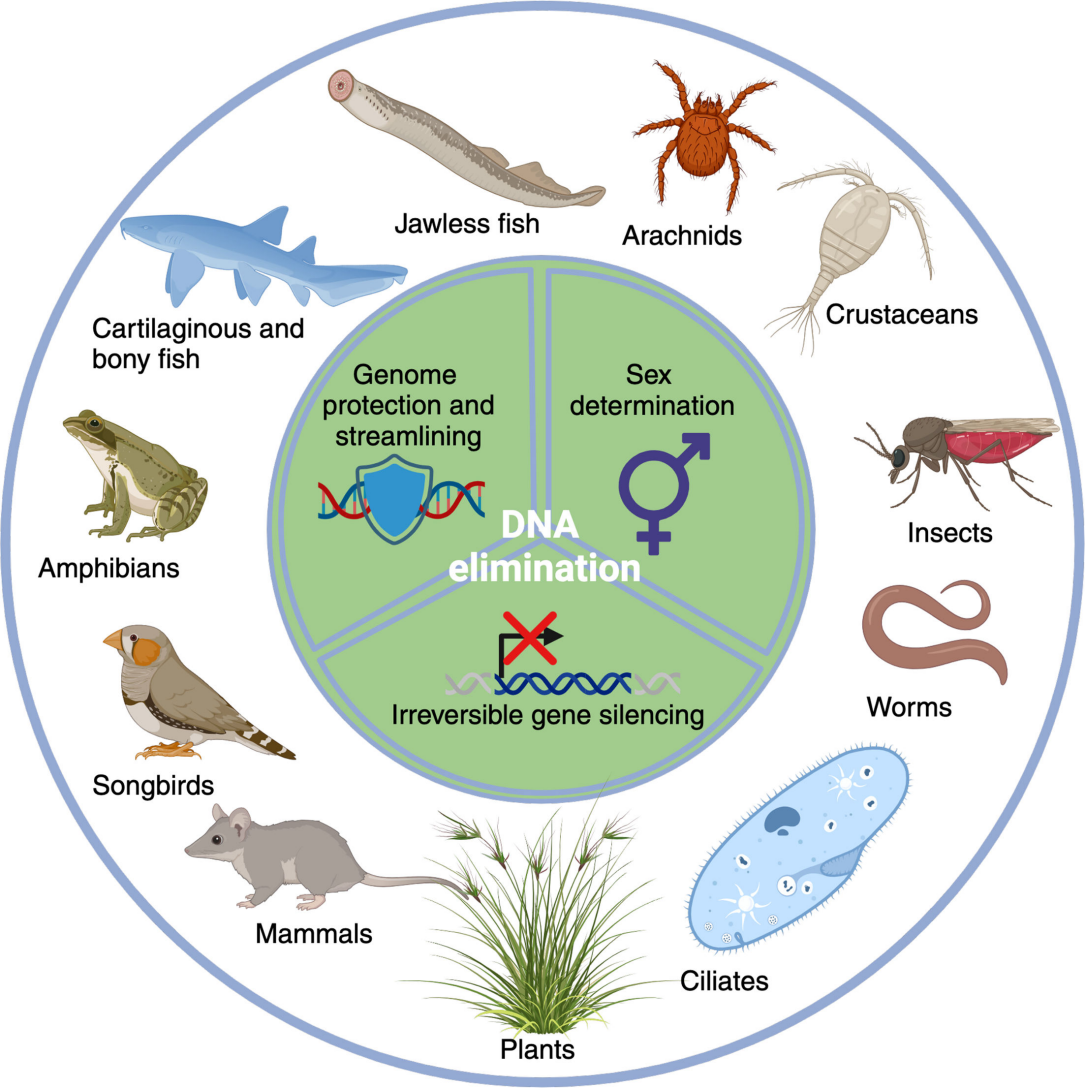
Diverse groups of eukaryotic organisms use programmed DNA elimination (and chromatin diminution) in their postmeiotic development. This includes the following genera: **Ciliates**: *Paramecium* [9], *Tetrahymena* [10], *Oxytricha* [10], *Stylonychia* [11], *Euplotes* [11]; **Worms**: *Strongyloides* [12], *Oscheius* [13], *Ascaris* [14], *Parascaris* [15]; **Insects**: *Phragmatobia* [8] (moths), *Liposcelis^12^* (booklice), *Bacillus* [16] (stick insects), *Bradysia* [17] and *Sciara* [8] (fungus gnats), *Mayetiola* [18] (hessian fly), Nasonia [19] (jewel wasp); **Copepods**: *Cyclops* [20], *Mesocyclops* [21]; **Arachnids**: *Metaseiulus* [22]; **Jawless fish**: *Petromyzon* [23] (lamprey), *Myxine* [24] (hagfish); **Fish**: *Hydrolagus* [25]*, Hypseleotris* [26]; **Amphibians**: Pelophylax [27], Bufotes [28]; **Songbirds**: *Taeniopygia* [29] *(zebra finch*), *Lonchura* [30]; **Mammals**: Perameles [31] and Isoodon [32] (bandicoots), Acomys [33] (spiny mouse); **Plants**: *Aegilops* [34] (goat grass), *Brachiaria* [35], *Hordeum* [36].

Surprisingly, another form of elimination exists, which leads to the programmed DNA elimination (PDE) of defined genomic regions. Notably, this process occurs reproducibly in each subsequent generation and as part of meiotic or postmeiotic embryonic development in a normal developmental cycle. PDE events are present across diverse clades along the tree of life including various metazoans and vertebrates [8] (Figure 1). While many molecular details about the process still remain elusive, it is clear that the known PDE events involve different machineries. This suggests that PDE evolved multiple times independently [37], suggesting that this convergent evolution [37] must have underlying evolutionary advantages. These include the possibility to irreversibly silence unnecessary genes, the ability to protect the genome from invasive sequences, or enable sex determination (Figure 1) from the same genomic material. Additionally, PDE can enable a similar degree of innovations as the products of alternative RNA splicing, which can generate novel protein products [38]. In this review, we aim to provide an overview of the known biological roles of programmed elimination of DNA in the various organisms, followed up by the molecular mechanisms that enable the discrimination of retained and eliminated sequences. We include the present state-of-the-art hypotheses and future directions for experimental validation. Specific focus is placed on the involvement of small RNAs (sRNAs) and the potential of such sRNA-guided molecular machinery for the development of novel tools for genome editing.

## Functions of PDE

In eukaryotes, the roles of PDE are diverse [39] and can be divided into three general groups: (i) sex determination and mating type or species compatibility; (ii) irreversible gene silencing for cell type differentiation; and (iii) genomic defense against invading sequences (Figure 1).

Sex determination based on chromosome segregation and elimination during embryogenesis is found in the booklice *Liposcelis*, with heterochromatin formation on the paternal chromosome followed by elimination [40]. PDE also enables sex determination in the roundworms from the genus *Strongyloides* [12]. In some plants, DNA elimination allows for species separation and determination of hybrid compatibility based on the loss or retention of paternal chromosomes [35,36]. Similar elimination of paternal genomes was observed in wasps [19] and arachnids [22]. Additionally, the whole paternal genome can be removed during gametogenesis in hybridizing species, [28] including frogs [27], insects [16,17], and fishes [25,26]. In mammals, various species also use PDE for the inactivation of sex chromosomes [41]. Marsupials eliminate the Y chromosome from specific cell lineages [32]. Additionally, the paternal X chromosome is eliminated in some female bandicoots, in a form of X-chromosomal dosage compensation [31]. An X0/XY sex chromosomal mosaicism is present in some rodents, like the spiny mouse [33], in which the male sexual chromosomes are eliminated from the somatic cells.

Genome streamlining is another prominent role of PDE. In jawless vertebrates like lampreys [23] and hagfish [24], the process targets both repetitive elements and also developmentally specific genes. The elimination enables an irreversible control mechanism of the embryogenesis transcriptional program and streamlines the genome content of the somatic cells. Differential chromosomal segregation during meiosis is a prominent form of PDE in thousands of songbird species that karyotypically differentiate their germline and somatic cells [29,30]. Notably, the indispensability of the songbird germline-restricted chromosome is likely due to a single highly conserved gene that is encoding for the RNA-binding protein CPEB1 involved in oocyte maturation [42]. Similarly, in the parasitic worm *Ascaris*, PDE has regulatory roles such as the permanent silencing of genes that are essential for gametogenesis and embryogenesis in somatic cells [14,15]. Recently, PDE was also discovered in *Oscheius tipulae*, a free-living member of the *Rhabditidae* nematodes to which also the common model organism *Caenorhabditis elegans* belongs, but the functional significance of the process for the organism remains to be determined [13]. Another form of genome streamlining called somatic mosaicism is formed by a selective elimination of DNA sequences in parts of an organism, where these genes are no longer necessary. This form of PDE has been detected in the roots of goat grass *Aegilops speltoides,* in which the B chromosome is eliminated [34]. Another prominent example for genome streamlining exists in unicellular organisms with a nuclear dimorphism that enables two distinct types of nuclei, a germline and a somatic nucleus, to coexist in a single cell [9]. The somatic genome in many ciliates is highly polyploid (e.g., in *Paramecium, n* = 1600) [11], thus streamlining its contents can significantly reduce the energetic costs for its replication. This DNA replication efficiency has been previously observed in other aquatic organisms [43].

In addition to genome streamlining by removing unnecessary content, the ability to selectively eliminate DNA can provide the means for a defense mechanism against the propagation of invasive sequences like transposons. These elements are particularly active during sexual reproduction in ciliates [44] and oogenesis in copepods [45]. Numerous eliminations of invasive DNA sequences are observed in ciliates, with some species removing as much as 97% of their germline genome [11]. In copepods, more than 80% of the germline genome consists of repetitive elements that are efficiently eliminated during embryogenesis [20,21]. The particular bias for the elimination of evolutionary younger and highly active transposon sequences suggests that PDE serves as a form of genome protection [21].

## Cellular mechanisms for discrimination of eliminated and retained DNA

To initiate any form of PDE, the cells must correctly recognize the DNA regions destined for elimination and retention. Due to the ancient origins of the process, and a convergent evolution, various mechanisms exist in different species. This is also due to the different lengths of the eliminated sequences ranging from small fragments to whole chromosomes.

**Figure 2:**
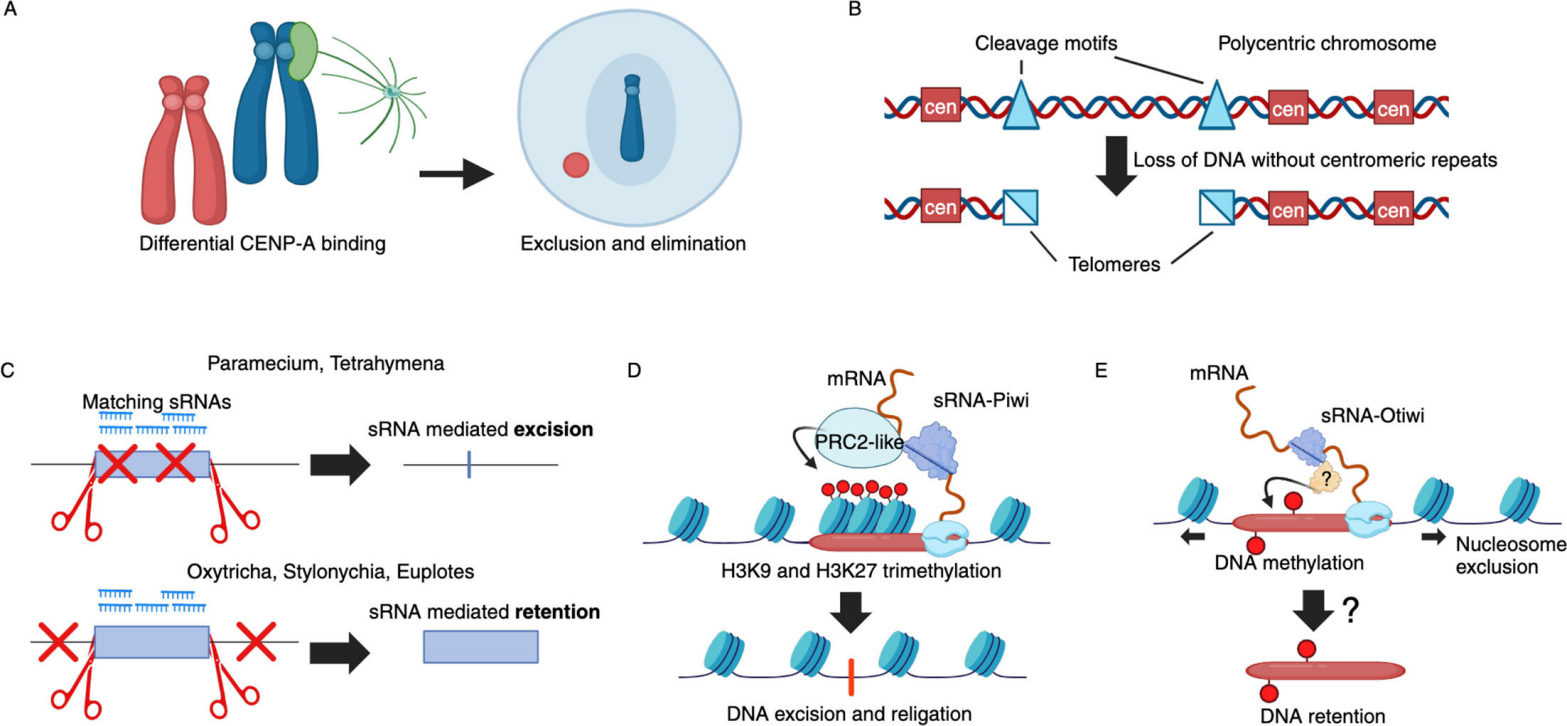
Differentiating DNA regions for elimination or retention. (**A**) Differential binding of centromeric proteins leads to retention of bound and exclusion of unbound chromosomes [36]. (**B**) DNA regions lacking centromeric repeats are lost during mitosis [46,47]. (**C**) In different ciliated organisms, the sRNAs could determine either the eliminated or the maintained sequences [9,11]. (**D**) sRNAs guide a PRC2-like complex for the deposition of H3K9me3, and H3K27me3 marks on the histones of eliminated transposable elements in ciliates [48,49]. (**E**) sRNA-guided methylation complex could potentially enable targeted sequence retention in ciliates [50,51]. sRNAs, small RNAs.

A simple mechanism for the selective elimination of whole chromosome is present in plant hybrids and involves the differential affinity of centromeric proteins to the parental centromeric repeats. The lack of centromeres on a chromosome leads to its elimination during subsequent mitosis [36] (Figure 2A). Similarly, the parasitic worms from the genus *Ascaris* eliminate large regions on their polycentric chromosomes that lack centromeric repeats [46]. These regions are flanked by conserved cleavage sites and upon excision excluded into micronuclei during mitosis [46,47] (Figure 2B). To retain these regions of the polycentric chromosomes in the germline, it is likely that the transcription of a specialized centromeric RNA is needed for RNA-mediated scaffolding that initiates the formation of a transient germline-specific centromeres, similar to the already described ones in *C. elegans* [52].

The precise elimination of short sequences requires more sophisticated demarcation mechanisms. Such PDE events are present in ciliates, and the eliminated sequences are often shorter than 100 bp [53] and lack long conserved sequence motifs [53,54]. Specific DNA recognition sequences typically allow a sequence-specific binding by proteins such as restriction nucleases or transposases [55]. Interestingly, the lack of conserved motives at the ends of eliminated sequences in ciliates stems from an sRNA-mediated specific recognition [56]. This form of delineation suggests base pairing interactions of the sRNAs similar to other sRNA-guided systems such as CRISPR and the recently described ragath-18-derived sRNAs that guide precise DNA targeting of IS607-encoded nucleases [57]. Notably, both positive (protection of sequences) and negative (marking for elimination) mechanisms exist in different ciliate species [11]. In *Paramecium* and *Tetrahymena*, sRNAs are complementary to the eliminated regions [9,10], whereas in *Oxytricha* and *Stylonychia*, the sRNAs mark the sequences that need to be retained [10,11] (Figure 2C). While sRNA-to-DNA base pairing has not yet been detected in ciliates, the PDE-specific sRNAs resemble piRNAs in their biogenesis and are loaded into PIWI-like proteins [58]. In *Paramecium*, the sRNA–PIWI complexes recognize TFIIS4-driven noncoding transcripts, which are produced in the new somatic nucleus before DNA elimination and thus contain eliminated sequences [59]. Interestingly, the proper execution of meiotic recombination is essential for the subsequent DNA elimination step, suggesting that a DNA development cycle with multiple checkpoints exists [60]. In line with this, at least two meiosis-specific factors, Spt5m and Spt4m, have been identified as regulators of transcription of the long noncoding RNAs, from which the initial guiding sRNAs are produced [61,62].

Similar to transposon silencing in other eukaryotes, the ciliate sRNA–protein complex binds to the complementary transcripts and then recruits effector proteins leading to heterochromatinization. Specifically, it has been shown that sRNA-loaded PIWI proteins can recruit a PRC2-like complex to establish H3K9me3 and H3K27me3 modifications at the sites of DNA destined for elimination [48,49] (Figure 2D). In the diatom *Phaeodactylum tricornutum,* sRNAs of similar length (26–31 bp) were recently found as essential for the establishment of the H3K9me3 and H3K27me3 repressive marks on transposable elements [63]. The similarity of this sRNA-guided heterochromatinization system of this diatom to the ciliate one suggests that it was probably present in the last common ancestor of the SAR clade and later repurposed or expanded in ciliates for genomic eliminations of the repressed regions.

Interestingly, heterochromatinization is also related to paternal genome elimination in some insects, since the eliminated genetic material accumulates heterochromatin-associated proteins before its elimination during meiosis [64,65]. The links between PDE and heterochromatin formation suggest that PDE probably evolved as a process for irreversible gene silencing. This is further corroborated by the discovery that many spermatogenesis genes in *C. elegans* subject to piRNA-mediated silencing [66] are permanently eliminated from the somatic cells of other worm species [13,67]. Similarly, the mammalian homologs of some eliminated genes in lampreys are silenced by PRC2-mediated heterochromatinization during embryonic development [68,69].

It must be noted that in ciliates, many of the eliminated fragments are actually smaller than the histone footprint [70]. The placement of nucleosomes and histone tail modifications is insufficient to explain the base–pair precision of the cleavage. This suggests that pleiotropic effects of the sRNA–PIWI complexes exist, leading on one side to heterochromatin formation on specific longer sequences akin to the canonical piRNA functions [71]. But, in the subset of eliminated sequences that require sRNA guidance, the sRNAs could also directly mark DNA for excision. Several reports suggest that in addition to histone modifications, direct nucleotide modifications are differentially deposited [50,51,72]. The role of such nucleotide modifications is presently unknown but could potentially regulate the nuclease activity (Figure 2E) similar to the toxin–antitoxin nuclease systems in bacteria. Proteins from these families form extensive defense systems against foreign nucleic acids, and the nuclease activity is controlled by RNA fragments and nucleotide modifications [73].

In lampreys, hypermethylation of cytosines is observed specifically on the eliminated sequences [72]. Similarly, DNA methylation presents a mechanism for suppression of transposable elements in plants. The target sequences are marked by complementary sRNAs that lead to sequence-dependent methylation of these regions in the germline [74]. The processing of the involved sRNAs requires Tudor domain Argonaute proteins [75], similar to the sRNA processing systems involved in PDE in ciliates [11]. Since PDE in ciliates also targets transposons and transposon remnants [76], it raises the possibility that sRNA-mediated DNA methylation marks in ciliates govern the elimination process. In line with such a hypothesis, a highly active eukaryotic DNA adenine methylation complex was discovered recently in *Tetrahymena* [50,77]. Furthermore, in *Paramecium* and *Oxytricha*, DNA 6-adenine methylation (6mA) has functional roles and disfavors nucleosome positioning on DNA [51,78] (Figure 2E). The disruption of the methylation complex causes significant lethality of the progeny that executed PDE [51]. Defects in the DNA elimination could also be caused by mispositioning of the nucleosomes, since correct nucleosome remodeling is required for DNA excision [79]. Additionally, the 6mA-modified nucleic acids could hypothetically allow RNA–DNA cross-talk during the sRNA-guided phase of PDE, similar to a recently discovered methylation-dependent transposable element suppression mechanism in human embryonic stem cells [80]. Further research into the DNA modification landscape of ciliates is needed to clarify the roles of nucleic acid modifications for the programmed genomic excisions.

## Molecular machinery involved in programmed DNA cleavage

**Figure 3:**
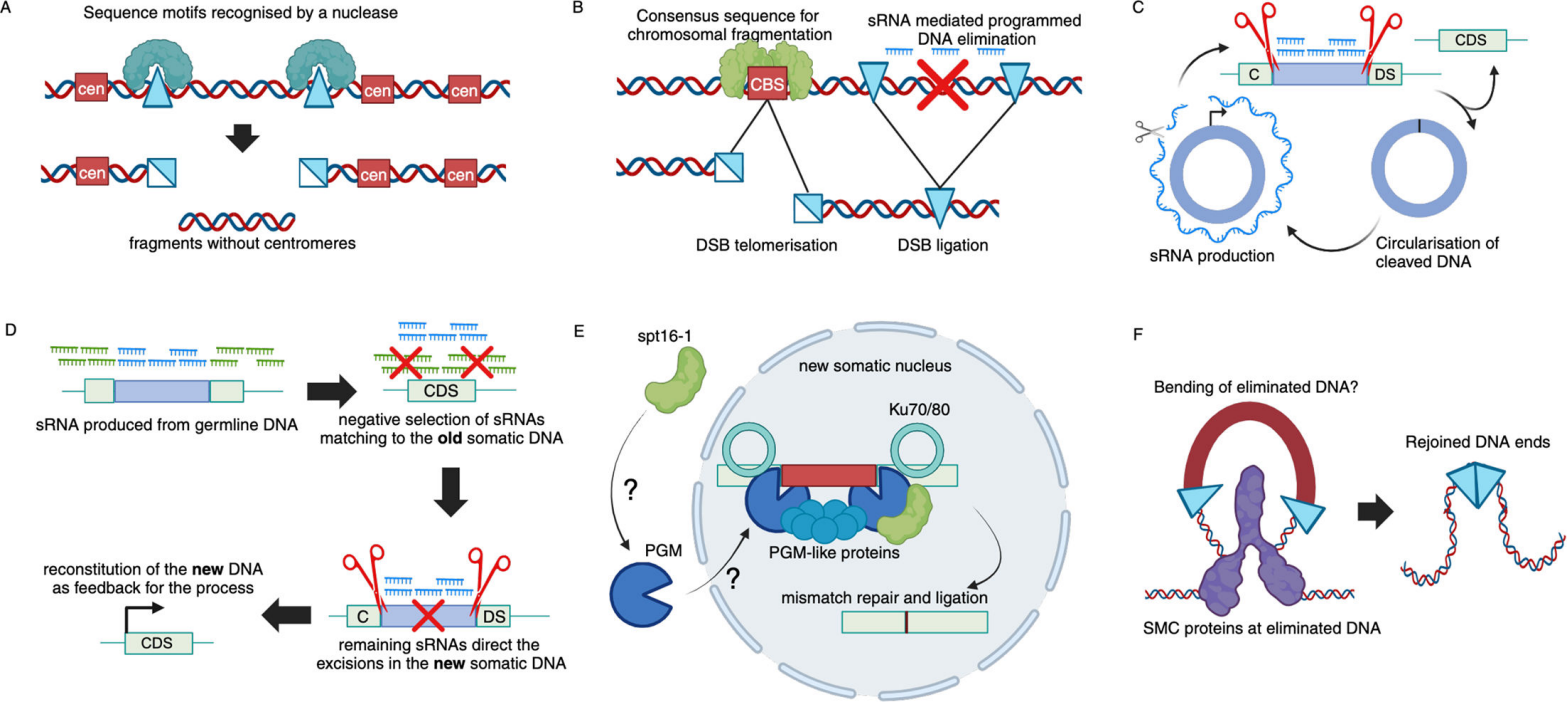
Molecular mechanics of different types of programmed DNA cleavage. (**A**) Conserved flanking motifs can serve for recognition by sequence specific nucleases [13]. (**B**) Chromosomal break sites (CBS) and sRNA-mediated DNA elimination can coexist, albeit the outcomes of the two processes are different with CBS resulting in *de novo* telomerized DNA ends [81,82]. (**C**) The excised DNA fragments are circularized and serve the production of sRNA precursor transcripts as a positive feedback loop for excision [83]. (**D**) In *Paramecium*, the guiding sRNAs are produced from the germline, but sRNAs matching to the old somatic nucleus are removed, before the remaining sRNA guide the excisions in the new somatic nucleus. The progress of the excision adds a feedback cross-talk to the old somatic and the germline nucleus, e.g., regulating gene expression there. (**E**) The PiggyBac-like nuclease (PGM) requires interactions with spt16-1 for import into the new developing somatic nucleus, where PGM in complex with PGM-like proteins and components of the NHEJ machinery conducts the genomic excisions [84–86]. (**F**) SMC proteins as part of a condensin complex could be involved in DNA looping that ensures that the cleaved DNA ends are in close proximity [87]. sRNA, small RNA.

Since the PDE process likely evolved multiple times separately, it is presently assumed that various enzymes and principles are involved in the cleavage step. In the worm *Oscheius tipulae*, the eliminated DNAs are flanked by a conserved sequence motif, which likely recruits the cleavage complex to the correct site [13] (Figure 3A). In ciliates, in addition to the previously mentioned sRNA-guided cleavage, an sRNA-independent cleavage coexists. The latter one serves likely for the elimination of centromeric regions [88] and chromosomal breakage [89], which has the function to split the germline chromosomes into smaller somatic chromosomes [81]. Chromosomal breakage is followed by *de novo* telomerization of the generated DNA ends [82] (Figure 3B). In contrast, sRNA-guided PDE usually leads to rejoining of the free DNA ends directly after cleavage, since this is required for the reconstitution of protein coding sequences (CDSs) interrupted by the eliminated DNA (Figure 3B). In *Oxytricha*, CDS reconstitution involves a comparison of the DNA fragments to long RNAs prior to DNA ligation in a process known as unscrambling [90]. This is necessary for reconstructing the correct order of the fragments as these are dispersed across the germline genome in both different orientation and succession [91,92]. The complex downstream processing of the free DNA ends is suggestive of a coupling of the DNA cleavage and rejoining. In *Paramecium*, most excisions occur within a single genome endoreplication, from a polyploidy of 32n to 64n [44]. However, the DNA removal process seems to occur sequentially, and two distinct classes of sRNAs are involved [83]. The initial scanRNAs are transcribed from the germline, whereas the subsequent iesRNAs are produced directly from the eliminated DNA, thereby creating a positive feedback loop for excisions [83] (Figure 3C).

This idea that PDE is a precisely executed multistep process is further supported by the fine-tuning of the gene expression of PDE-related genes in *Paramecium*, which is governed by the progression of the PDE process [84]. A nuclear cross-talk in the opposite direction is already necessary for the initial selection of the sRNAs [93] (Figure 3D), and likely the bi-directional cross-talk enables checkpoints during the process and the transition from a germline to somatic nuclear phenotype. While the germline nucleus produces the transcripts from which the sRNAs are produced, it is considered otherwise transcriptionally inactive [94]. However, germline-limited sequences can be expressed during the development of the somatic nucleus as recently reported for an essential PiggyBac transposon-derived gene [95]. Typically, such PiggyBac transposases recognize long inverted terminal repeats (ITRs) [55], which are not present in the eliminated sequences in ciliates, as these are usually shorter than the canonical ITRs. Nonetheless, in some ciliate species such as *Paramecium* and *Tetrahymena*, a domesticated PiggyBac-like transposase executes the DNA cleavage step [96]. While a knockdown of the enzyme causes a complete retention of eliminated sequences [96], the selective nuclease activity has not been directly demonstrated for purified protein. This discrepancy probably stems from nucleolytic licensing through additional factors in the excision complex. These include scaffolding catalytically inactive PiggyBac-like proteins [97] and components linked to DNA repair (Ku70/80) [85,98] and ligation (Ligase IV) [86], whose knockdown prevented DNA cleavage (Figure 3E). Noteworthy, the importance of the interactions for nucleolytic activity was demonstrated using a mutant Ku70 that lacks DNA affinity but can still interact with the nuclease. Expression of this protein re-enabled cleavage in wt Ku70 knockdown cells and also resulted in a massive increase of incorrectly rejoined chromosomes and CDS-internal telomeres [85].

The involvement of additional enzymes in the excision is likely. An RNA helicase is essential for establishing the base pairing between sRNAs and the targeted transcripts in *Tetrahymena* [99], a closely related organism to *Paramecium*, in which the excisions are also conducted by a domesticated PiggyBac transposon. An additional factor in the process is the histone chaperone spt16-1, which enables the proper localization of the PiggyBac to the developing nucleus [100]. It would be interesting to understand if this is based on direct interactions with the cleavage complex or through exchange of nucleosome subunits corresponding to the established function of spt16 chaperones. The proper localization and recruitment of the excision complex could also be controlled by specific posttranslational modifications. One such example is SUMOylation, which is an essential regulatory process involved in sRNA-mediated transposon silencing and heterochromatinization [101]. This posttranscriptional modification is widespread also in ciliates and specifically up-regulated during PDE [101]. Furthermore, SUMOylation can be PIWI-dependent [102] and linked to piRNA-mediated heterochromatinization of transposable elements as reported in Drosophila, where Panoramix recruits heterochromatinization factors when SUMOylated [103].

Finally, a specific DNA organization might be necessary for DNA elimination and correct joining of the free DNA ends (Figure 3F). This is corroborated by the recent finding of a meiosis-specific SMC protein and the condensin complex in DNA elimination in *Paramecium* [87]. Further investigation needs to clarify whether the complex enables developmental-specific gene expression or is required for the formation of DNA loops recognized by the cleavage machinery (Figure 3F). An example for such functions is the synaptonemal complex in *C. elegans*, which is formed by similar components as the PDE machinery in ciliates, namely Argonaute proteins, sRNAs, and a meiosis-specific SMC-1 [104]. Furthermore, the involvement of condensins in suppression of the LINE-1 retrotransposons [105] has been reported recently. Silencing in higher eukaryotes has paralleled to the excision of transposons in ciliates, further highlighting the potential function of condensins as facilitators of genomic excisions.

## Outlook: the potential of the DNA elimination machinery for genome editing

In most eukaryotes, piRNA-like sRNAs orchestrate the cellular response to invading transposable elements [71,106]. In ciliates, this suppression response is driven to the extreme, resulting in the permanent elimination of transposon-derived sequences from their somatic genome. This is possible due to a nuclear dimorphism, which allows for a comparison of the contents of both nuclei, in a process known as scanning. Any newly appearing DNA can therefore be efficiently suppressed. Similar to piRNA-mediated transposon silencing in other eukaryotes, the excision process in ciliates involves two separate waves of sRNAs. The secondary sRNAs are produced directly from newly excised DNA fragments in a positive feedback loop [83] (Figure 3C). Because most of these DNA fragments are smaller than 100 bp, their transcription requires initial concatemerization and circularization to longer DNA molecules that can be transcribed [107]. This unorthodox solution exemplifies a general trend of ciliates to acquire and repurpose molecular machinery for eccentric biological properties [108,109]. Understanding such unconventional machineries is an essential step toward the ability to integrate them into engineered synthetic biology systems [110]. Engineered systems hold promise both for the development of futuristic biotechnological manufacturing methods [111] but also for establishing novel therapies for complex diseases [112].

The PiggyBac transposon suppression system in ciliates is an example for the significant evolutionary advancements achieved by transposon domestication. This process has been a prominent driver of evolution as evidenced by the adaptive immune system developed from an ancient transposase in vertebrates [113] and the placental development in mammals [9]. Additionally, the prokaryotic CRISPR-Cas system originated from RNA-guided transposon-derived proteins [114] similar to the Fanzor nucleases in eukaryotes [115–117]. Interestingly, it is hypothesized that the ability of ciliates to control transposable elements represents a double-edged sword. By limiting the detrimental consequences of transposon invasion, it also fostered the propagation of transposons in the germline [76,118]. This in term favored an evolution of the genome of the host organism [9] facilitated by the additional raw sequence material.

**Figure 4:**
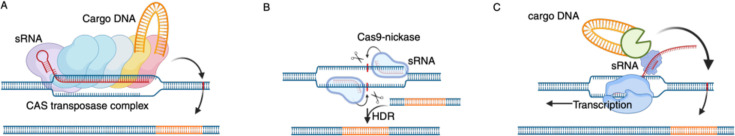
sRNA-guided tools for high-precision-targeted insertions of cargo DNA. (**A**) Type I CRISPR-associated transposase (CAST) complex for RNA-guided genetic insertions [122–124]. (**B**) Cas9-nickase enzyme with dual-guide RNA to achieve a double-stranded break [125]. (**C**) A nascent transcript binding transposase complex corresponding to the ciliate genome excision complex [9,97]. sRNA, small RNA.

Noteworthy, the eukaryotic transposases from the PiggyBac family are among the most efficient molecular tools for genomic integration of long transgenes in human cells [119]. Vertebrate genomes encode many additional highly active transposases that could also be harnessed for genome editing [120]. However, a major obstacle for their use at a clinical setting stems from the safety concerns due to the uncontrollable localization of the insertions [121]. Consequently, RNA-guided transposases could enable efficient integration of DNA cargo into specific target sites, as exemplified by the bacterial transposases derived from omega and Tn7 CRISPR-associated transposons (CAST) (Figure 4A). Type I CASTs (evolved from Tn7 transposons) have recently allowed for an insertion of genetic cargo at locations that are defined by RNA guides into cyanobacteria [126] and human cells [122]. However, due to the complexity of the machinery, further improvements in efficiency are needed. Alternatively, type V-K CASTs (evolved from Tn5053 transposons) are more efficient but often result in cointegration of undesirable sequences such as the plasmid backbone through replicative instead of cut-and-paste transposition [127]. Using engineering approaches a fusion protein of a type V-K CAST with a homing DNA nickase allowed for single-product insertions [123]. Usage of dual-nickase activity was also employed previously with the aim of reducing off-target activity of the Cas9 nuclease, as it allows for using two separate guide RNAs for the cleavage at the correct target site [125] (Figure 4B). Notably, it was recently reported that negative DNA supercoiling leads to striking increase in off-target binding by Cas enzymes [128]. A possibility to bypass the effects of DNA topology on binding specificity can be imagined through an indirect recruitment to the desired DNA location via binding to nascent transcripts using sRNA/PIWIs, like in some ciliates (Figure 4C). For achieving targeted insertion at this site, the integration activity of the transposase proteins from ciliates has to be restored through protein engineering. Thus, whether the sRNA-guided DNA elimination machinery including PiggyBac-like transposases meets all requirements for use for precise genome editing in eukaryotes, such as humans, remains to be further investigated. A better understanding of naturally occurring genome editing processes such as PDE across various life forms, and not only in ciliates, will likely augment the list of available genome editing tools.

**Perspectives**

- Importance of the field: Programmed DNA elimination (PDE) is a strikingly prevalent process in eukaryotes. It has diverse roles in both simple and complex organisms. Notably, the wide distribution is the product of a convergent evolution. This highlights the importance of the underlying challenge leading to this development: what to do with unnecessary DNA? While in some organisms the unnecessary portions of DNA are compacted and suppressed, it seems that eliminating it altogether is also an efficient option. In the specific case of ciliates, the PDE was perfectioned in terms of both processivity and precision allowing the elimination of thousands of fragments with base-pair precision.
- Current thinking: The precision of DNA elimination in ciliates allows it to serve as a form of immune system that protects the organisms against invasive DNA sequences such as transposons. The process involves an RNA-based comparison of the gene contents of the germline and somatic genomes during sexual reproduction. As a first step, small RNAs (sRNAs) are produced from the germline. Those that do not find a match in the correct old version of the somatic genome are transferred to the newly forming somatic nucleus during its formation from the germline sequence. The sRNAs are only complementary to sequences that appear in the germline but not in the old somatic nucleus. Through a complex and presently mechanistically enigmatic cascade, the sRNAs then guide the elimination of their matching DNA sequences.
- Future directions: The functions of PDE in the suppression of invasive sequences could offer insights into the early evolutionary stages of eukaryotic chromatin. Additionally, advanced proteomic techniques developed in the recent years will allow to probe the sRNA-directed DNA elimination process in ciliates in mechanistic detail. Understanding the link between the specific DNA recognition by sRNAs and the subsequent DNA elimination step holds the potential to uncover new molecular tools for the manipulation of DNA *in vivo*. Such tools could be then repurposed for expanding the available gene editing toolbox. Safer and more efficient DNA editing systems are essential for the advancement of such methods toward therapeutic purposes and clinical implementation.

## Abbreviations

CAST: CRISPR-associated transposase
CDSs: protein coding sequences
ITRs: inverted terminal repeats
PDE: programmed DNA elimination
sRNAs: small RNAs

## References

[1] Cortés-Ciriano I., Lee J.J.K., Xi R., Jain D., Jung Y.L., Yang L. (2020). Comprehensive analysis of chromothripsis in 2,658 human cancers using whole-genome sequencing. Nat. Genet..

[2] Bakhoum S.F., Cantley L.C (2018). The multifaceted role of chromosomal instability in cancer and its microenvironment. Cell.

[3] Shoshani O., Brunner S.F., Yaeger R., Ly P., Nechemia-Arbely Y., Kim D.H. (2021). Chromothripsis drives the evolution of gene amplification in cancer. Nature.

[4] Bakhoum S.F., Ngo B., Laughney A.M., Cavallo J.A., Murphy C.J., Ly P. (2018). Chromosomal instability drives metastasis through a cytosolic DNA response. Nature.

[5] Crasta K., Ganem N.J., Dagher R., Lantermann A.B., Ivanova E.V., Pan Y. (2012). DNA breaks and chromosome pulverization from errors in mitosis. Nature.

[6] Domingo-Muelas A., Skory R.M., Moverley A.A., Ardestani G., Pomp O., Rubio C (2023). Human embryo live imaging reveals nuclear DNA shedding during blastocyst expansion and biopsy. Cell.

[7] Gruhn J.R., Zielinska A.P., Shukla V., Blanshard R., Capalbo A., Cimadomo D. (2019). Chromosome errors in human eggs shape natural fertility over reproductive life span. Science.

[8] Wang J., Davis R.E (2014). Programmed DNA elimination in multicellular organisms. Curr. Opin. Genet. Dev..

[9] Stefanov B.A., Nowacki M (2024). Epigenetics in Biological Communication.

[10] Allen S.E., Nowacki M (2020). Roles of noncoding RNAs in ciliate genome architecture. J. Mol. Biol..

[11] Rzeszutek I., Maurer-Alcalá X.X., Nowacki M (2020). Programmed genome rearrangements in ciliates. Cell. Mol. Life Sci..

[12] Streit A., Wang J., Kang Y., Davis R.E (2016). Gene silencing and sex determination by programmed DNA elimination in parasitic nematodes. Curr. Opin. Microbiol..

[13] Dockendorff T.C., Estrem B., Reed J., Simmons J.R., Zadegan S.B., Zagoskin M.V (2022). The nematode *Oscheius tipulae* as a genetic model for programmed DNA elimination. Curr. Biol..

[14] Wang J., Mitreva M., Berriman M., Thorne A., Magrini V., Koutsovoulos G (2012). Silencing of germline-expressed genes by DNA elimination in somatic cells. Dev. Cell.

[15] Müller F., Tobler H (2000). Chromatin diminution in the parasitic nematodes ascaris suum and parascaris univalens. Int. J. Parasitol..

[16] Forni G., Mantovani B., Mikheyev A.S., Luchetti A (2024). Parthenogenetic stick insects exhibit signatures of preservation in the molecular architecture of male reproduction. Genome Biol. Evol..

[17] Hodson C.N., Jaron K.S., Gerbi S., Ross L (2022). Gene-rich germline-restricted chromosomes in black-winged fungus gnats evolved through hybridization. Plos Biol..

[18] Crane Y.M., Crane C.F., Cambron S.E., Springmeyer L.J., Schemerhorn B.J (2023). Molecular characterization of eliminated chromosomes in hessian fly (Mayetiola destructor (Say)). Chromosome Res..

[19] Lee H., Seo P., Teklay S., Yuguchi E., Benetta E.D., Werren J.H. (2023). Ability of a selfish B chromosome to evade genome elimination in the jewel wasp, *Nasonia vitripennis*. Heredity.

[20] Degtyarev S., Boykova T., Grishanin A., Belyakin S., Rubtsov N., Karamysheva T. (2004). The molecular structure of the DNA fragments eliminated during chromatin diminution in *Cyclops kolensis*. Genome Res..

[21] Sun C., Wyngaard G., Walton D.B., Wichman H.A., Mueller R.L (2014). Billions of basepairs of recently expanded, repetitive sequences are eliminated from the somatic genome during copepod development. BMC Genomics.

[22] Hoy M.A., Waterhouse R.M., Wu K., Estep A.S., Ioannidis P., Palmer W.J. (2016). Genome sequencing of the phytoseiid predatory mite metaseiulus occidentalis reveals completely atomized hox genes and superdynamic intron evolution. Genome Biol. Evol..

[23] Smith J.J., Antonacci F., Eichler E.E., Amemiya C.T (2009). Programmed loss of millions of base pairs from a vertebrate genome. Proc. Natl. Acad. Sci. U.S.A..

[24] Marlétaz F., Timoshevskaya N., Timoshevskiy V.A., Parey E., Simakov O., Gavriouchkina D. (2024). The hagfish genome and the evolution of vertebrates. Nature.

[25] Stanley H.P., Kasinsky H.E., Bols N.C (1984). Meiotic chromatin diminution in a vertebrate, the holocephalan fish *Hydrolagus collie* (Chondrichthyes, Holocephali). Tissue Cell.

[26] Schmidt D.J., Bond N.R., Adams M., Hughes J.M (2011). Cytonuclear evidence for hybridogenetic reproduction in natural populations of the Australian carp gudgeon (Hypseleotris: Eleotridae). Mol. Ecol..

[27] Chmielewska M., Dedukh D., Haczkiewicz K., Rozenblut-Kościsty B., Kaźmierczak M., Kolenda K. (2018). The programmed DNA elimination and formation of micronuclei in germ line cells of the natural hybridogenetic water frog *Pelophylax esculentus*. Sci. Rep..

[28] Stöck M., Ustinova J., Lamatsch D.K., Schartl M., Perrin N., Moritz C (2010). A vertebrate reproductive system involving three ploidy levels: hybrid origin of triploids in a contact zone of diploid and tetraploid palearctic green toads (*Bufo viridis* subgroup). Evolution.

[29] Borodin P., Chen A., Forstmeier W., Fouché S., Malinovskaya L., Pei Y. (2022). Mendelian nightmares: the germline-restricted chromosome of songbirds. Chromosome Res..

[30] Sotelo-Muñoz M., Poignet M., Albrecht T., Kauzál O., Dedukh D., Schlebusch S.A. (2022). Germline-restricted chromosome shows remarkable variation in size among closely related passerine species. Chromosoma.

[31] Johnston P.G., Watson C.M., Adams M., Paull D.J (2002). Sex chromosome elimination, X chromosome inactivation and reactivation in the southern brown bandicoot Isoodon obesulus (Marsupialia: Peramelidae). Cytogenet. Genome Res..

[32] Watson C.M., Margan S.H., Johnston P.G (1998). Sex-chromosome elimination in the bandicoot isoodon macrourus using Y-linked markers. Cytogenet. Cell Genet..

[33] Castiglia R., Makundi R., Corti M (2007). The origin of an unusual sex chromosome constitution in *Acomys sp.* (Rodentia, Muridae) from Tanzania. Genetica.

[34] Ruban A., Schmutzer T., Wu D.D., Fuchs J., Boudichevskaia A., Rubtsova M. (2020). Supernumerary B chromosomes of *Aegilops speltoides* undergo precise elimination in roots early in embryo development. Nat. Commun..

[35] Ricci G.C.L., Pagliarini M.S., Valle C.B (2010). Genome elimination during microsporogenesis in two pentaploid accessions of *Brachiaria decumbens* (Poaceae). Genet. Mol. Res..

[36] Sanei M., Pickering R., Kumke K., Nasuda S., Houben A (2011). Loss of centromeric histone H3 (CENH3) from centromeres precedes uniparental chromosome elimination in interspecific barley hybrids. Proc. Natl. Acad. Sci. U.S.A..

[37] Kloc M., Kubiak J.Z., Ghobrial R.M (2022). Natural genetic engineering: a programmed chromosome/DNA elimination. Dev. Biol..

[38] Catania F., Schmitz J (2015). On the path to genetic novelties: insights from programmed DNA elimination and RNA splicing. Wiley Interdiscip. Rev. RNA.

[39] Zagoskin M.V., Wang J (2021). Programmed DNA elimination: silencing genes and repetitive sequences in somatic cells. Biochem. Soc. Trans..

[40] Hodson C.N., Hamilton P.T., Dilworth D., Nelson C.J., Curtis C.I., Perlman S.J (2017). Paternal genome elimination in *Liposcelis* booklice (Insecta: Psocodea). Genetics.

[41] Smith J.J., Timoshevskiy V.A., Saraceno C (2021). Programmed DNA elimination in vertebrates. Annu. Rev. Anim. Biosci..

[42] Schlebusch S.A., Rídl J., Poignet M., Ruiz-Ruano F.J., Reif J., Pajer P. (2023). Rapid gene content turnover on the germline-restricted chromosome in songbirds. Nat. Commun..

[43] Swan B.K., Tupper B., Sczyrba A., Lauro F.M., Martinez-Garcia M., González J.M. (2013). Prevalent genome streamlining and latitudinal divergence of planktonic bacteria in the surface ocean. Proc. Natl. Acad. Sci. U.S.A..

[44] Zangarelli C., Arnaiz O., Bourge M., Gorrichon K., Jaszczyszyn Y., Mathy N. (2022). Developmental timing of programmed DNA elimination in *Paramecium tetraurelia* recapitulates germline transposon evolutionary dynamics. Genome Res..

[45] Dedukh D., Krasikova A (2022). Delete and survive: strategies of programmed genetic material elimination in eukaryotes. Biol. Rev. Camb. Philos. Soc..

[46] Kang Y., Wang J., Neff A., Kratzer S., Kimura H., Davis R.E (2016). Differential chromosomal localization of centromeric histone CENP-A contributes to nematode programmed DNA elimination. Cell Rep..

[47] Carlton P.M., Davis R.E., Ahmed S (2022). Nematode chromosomes. Genetics.

[48] Xu J., Zhao X., Mao F., Basrur V., Ueberheide B., Chait B.T. (2021). A polycomb repressive complex is required for RNAi-mediated heterochromatin formation and dynamic distribution of nuclear bodies. Nucleic Acids Res..

[49] Miró-Pina C., Charmant O., Kawaguchi T., Holoch D., Michaud A., Cohen I (2022). Paramecium polycomb repressive complex 2 physically interacts with the small RNA-binding PIWI protein to repress transposable elements. Dev. Cell.

[50] Wang Y., Sheng Y., Liu Y., Pan B., Huang J., Warren A. (2017). N 6 -methyladenine DNA modification in the unicellular eukaryotic organism *Tetrahymena thermophila*. Eur. J. Protistol..

[51] Beh L.Y., Debelouchina G.T., Clay D.M., Thompson R.E., Lindblad K.A., Hutton E.R (2019). Identification of a DNA N6-adenine methyltransferase complex and its impact on chromatin organization. Cell.

[52] Gassmann R., Rechtsteiner A., Yuen K.W., Muroyama A., Egelhofer T., Gaydos L. (2012). An inverse relationship to germline transcription defines centromeric chromatin in *C. elegans*. Nature.

[53] Arnaiz O., Mathy N., Baudry C., Malinsky S., Aury J.M., Denby Wilkes C. (2012). The *Paramecium germline* genome provides a niche for intragenic parasitic DNA: evolutionary dynamics of internal eliminated sequences. Plos Genet..

[54] Catania F., McGrath C.L., Doak T.G., Lynch M (2013). Spliced DNA sequences in the *Paramecium germline*: their properties and evolutionary potential. Genome Biol. Evol..

[55] Chen Q., Luo W., Veach R.A., Hickman A.B., Wilson M.H., Dyda F (2020). Structural basis of seamless excision and specific targeting by piggyBac transposase. Nat. Commun..

[56] Chalker D.L., Fuller P., Yao M.C (2005). Communication between parental and developing genomes during tetrahymena nuclear differentiation is likely mediated by homologous RNAs. Genetics.

[57] Ren K., Zhou F., Zhang F., Yin M., Zhu Y., Wang S. (2024). Discovery and structural mechanism of DNA endonucleases guided by RAGATH-18-derived RNAs. Cell Res..

[58] Furrer D.I., Swart E.C., Kraft M.F., Sandoval P.Y., Nowacki M (2017). Two sets of Piwi proteins are involved in distinct sRNA pathways leading to elimination of germline-specific DNA. Cell Rep..

[59] Maliszewska-Olejniczak K., Gruchota J., Gromadka R., Denby Wilkes C., Arnaiz O., Mathy N. (2015). TFIIS-dependent non-coding transcription regulates developmental genome rearrangements. Plos Genet..

[60] Rzeszutek I., Swart E.C., Pabian-Jewuła S., Russo A., Nowacki M (2022). Early developmental, meiosis-specific proteins - Spo11, Msh4-1, and Msh5 - affect subsequent genome reorganization in *Paramecium tetraurelia*. Biochim. Biophys. Acta Mol. Cell Res..

[61] Gruchota J., Denby Wilkes C., Arnaiz O., Sperling L., Nowak J.K (2017). A meiosis-specific Spt5 homolog involved in non-coding transcription. Nucleic Acids Res..

[62] Owsian D., Gruchota J., Arnaiz O., Nowak J.K (2022). The transient Spt4-Spt5 complex as an upstream regulator of non-coding RNAs during development. Nucleic Acids Res..

[63] Grypioti E., Richard H., Kryovrysanaki N., Jaubert M., Falciatore A., Verret F. (2024). Dicer-dependent heterochromatic small RNAs in the model diatom species *Phaeodactylum tricornutum*. New Phytol..

[64] Tang X.F., Huang Y.H., Sun Y.F., Zhang P.F., Huo L.Z., Li H.S. (2023). The transcriptome of Icerya aegyptiaca (Hemiptera: Monophlebidae) and comparison with neococcoids reveal genetic clues of evolution in the scale insects. BMC Genomics.

[65] Ross L., Pen I., Shuker D.M (2010). Genomic conflict in scale insects: the causes and consequences of bizarre genetic systems. Biol. Rev. Camb. Philos. Soc..

[66] Cornes E., Bourdon L., Singh M., Mueller F., Quarato P., Wernersson E. (2022). piRNAs initiate transcriptional silencing of spermatogenic genes during *C. elegans* germline development. Dev. Cell.

[67] Wang J., Gao S., Mostovoy Y., Kang Y., Zagoskin M., Sun Y. (2017). Comparative genome analysis of programmed DNA elimination in nematodes. Genome Res..

[68] Smith J.J., Timoshevskaya N., Ye C., Holt C., Keinath M.C., Parker H.J. (2018). The sea lamprey germline genome provides insights into programmed genome rearrangement and vertebrate evolution. Nat. Genet..

[69] Saraceno C., Timoshevskiy V.A., Smith J.J (2024). Functional analyses of the polycomb-group genes in sea lamprey embryos undergoing programmed DNA loss. J. Exp. Zool. B Mol. Dev. Evol..

[70] Balan T., Lerner L.K., Holoch D., Duharcourt S (2024). Small-RNA-guided histone modifications and somatic genome elimination in ciliates. Wiley Interdiscip. Rev. RNA.

[71] Wang X., Ramat A., Simonelig M., Liu M.F (2023). Emerging roles and functional mechanisms of PIWI-interacting RNAs. Nat. Rev. Mol. Cell Biol..

[72] Angeloni A., Fissette S., Kaya D., Hammond J.M., Gamaarachchi H., Deveson I.W. (2024). Extensive DNA methylome rearrangement during early lamprey embryogenesis. Nat. Commun..

[73] Bell R.T., Sahakyan H., Makarova K.S., Wolf Y.I., Koonin E.V (2024). CoCoNuTs are a diverse subclass of type IV restriction systems predicted to target RNA. Elife.

[74] Chow H.T., Mosher R.A (2023). Small RNA-mediated DNA methylation during plant reproduction. Plant Cell.

[75] Takei T., Tsukada M., Tamura K., Hara-Nishimura I., Fukao Y., Kurihara Y. (2024). ARGONAUTE1-binding tudor domain proteins function in small interfering RNA production for RNA-directed DNA methylation. Plant Physiol..

[76] Sellis D., Guérin F., Arnaiz O., Pett W., Lerat E., Boggetto N. (2021). Massive colonization of protein-coding exons by selfish genetic elements in *Paramecium germline* genomes. Plos Biol..

[77] Yan J., Liu F., Guan Z., Yan X., Jin X., Wang Q. (2023). Structural insights into DNA N6-adenine methylation by the MTA1 complex. Cell Discov..

[78] Hardy A., Matelot M., Touzeau A., Klopp C., Lopez-Roques C., Duharcourt S. (2021). DNAModAnnot: a R toolbox for DNA modification filtering and annotation. Bioinformatics.

[79] Singh A., Häußermann L., Emmerich C., Nischwitz E., Seah B.K.B., Butter F. (2025). ISWI1 complex proteins facilitate developmental genome editing in *Paramecium*. Genome Res..

[80] Sun T., Xu Y., Xiang Y., Ou J., Soderblom E.J., Diao Y (2023). Crosstalk between RNA m6A and DNA methylation regulates transposable element chromatin activation and cell fate in human pluripotent stem cells. Nat. Genet..

[81] Klobutcher L.A., Gygax S.E., Podoloff J.D., Vermeesch J.R., Price C.M., Tebeau C.M. (1998). Conserved DNA sequences adjacent to chromosome fragmentation and telomere addition sites in *Euplotes crassus*. Nucleic Acids Res..

[82] Bétermier M., Klobutcher L.A., Orias E (2023). Programmed chromosome fragmentation in ciliated protozoa: multiple means to chromosome ends. Microbiol. Mol. Biol. Rev..

[83] Sandoval P.Y., Swart E.C., Arambasic M., Nowacki M (2014). Functional diversification of dicer-like proteins and small RNAs required for genome sculpting. Dev. Cell.

[84] Bazin-Gélis M., Eleftheriou E., Zangarelli C., Lelandais G., Sperling L., Arnaiz O. (2023). Inter-generational nuclear crosstalk links the control of gene expression to programmed genome rearrangement during the paramecium sexual cycle. Nucleic Acids Res..

[85] Bischerour J., Arnaiz O., Zangarelli C., Régnier V., Iehl F., Ropars V (2024). Uncoupling programmed DNA cleavage and repair scrambles the *Paramecium* somatic genome. Cell Rep..

[86] Kapusta A., Matsuda A., Marmignon A., Ku M., Silve A., Meyer E. (2011). Highly precise and developmentally programmed genome assembly in *Paramecium* requires ligase IV-dependent end joining. Plos Genet..

[87] Zhang F., Bechara S., Nowacki M (2024). Structural maintenance of chromosomes (SMC) proteins are required for DNA elimination in *Paramecium*. Life Sci. Alliance.

[88] Lhuillier-Akakpo M., Guérin F., Frapporti A., Duharcourt S (2016). DNA deletion as a mechanism for developmentally programmed centromere loss. Nucleic Acids Res..

[89] McDaniel S.L., Zweifel E., Harris P.K.W., Yao M.C., Cole E.S., Chalker D.L (2016). DRH1, a p68-related RNA helicase gene, is required for chromosome breakage in *Tetrahymena*. Biol. Open.

[90] Nowacki M., Vijayan V., Zhou Y., Schotanus K., Doak T.G., Landweber L.F (2008). RNA-mediated epigenetic programming of a genome-rearrangement pathway. Nature.

[91] Mochizuki K (2010). DNA rearrangements directed by non-coding RNAs in ciliates. Wiley Interdiscip. Rev. RNA.

[92] Nowacki M., Shetty K., Landweber L.F (2011). RNA-mediated epigenetic programming of genome rearrangements. Annu. Rev. Genomics Hum. Genet..

[93] Lepère G., Bétermier M., Meyer E., Duharcourt S (2008). Maternal noncoding transcripts antagonize the targeting of DNA elimination by scanRNAs in *Paramecium tetraurelia*. Genes Dev..

[94] Katz L.A (2001). Evolution of nuclear dualism in ciliates: a reanalysis in light of recent molecular data. Int. J. Syst. Evol. Microbiol..

[95] Feng L., Wang G., Hamilton E.P., Xiong J., Yan G., Chen K. (2017). A germline-limited piggyBac transposase gene is required for precise excision in *Tetrahymena genome* rearrangement. Nucleic Acids Res..

[96] Baudry C., Malinsky S., Restituito M., Kapusta A., Rosa S., Meyer E. (2009). PiggyMac, a domesticated piggyBac transposase involved in programmed genome rearrangements in the ciliate *Paramecium tetraurelia*. Genes Dev..

[97] Bischerour J., Bhullar S., Denby Wilkes C., Régnier V., Mathy N., Dubois E. (2018). Six domesticated PiggyBac transposases together carry out programmed DNA elimination in *Paramecium*. Elife.

[98] Marmignon A., Bischerour J., Silve A., Fojcik C., Dubois E., Arnaiz O. (2014). Ku-mediated coupling of DNA cleavage and repair during programmed genome rearrangements in the ciliate *Paramecium tetraurelia*. Plos Genet..

[99] Aronica L., Bednenko J., Noto T., DeSouza L.V., Siu K.W.M., Loidl J. (2008). Study of an RNA helicase implicates small RNA-noncoding RNA interactions in programmed DNA elimination in *Tetrahymena*. Genes Dev..

[100] Vanssay A., Touzeau A., Arnaiz O., Frapporti A., Phipps J., Duharcourt S (2020). The *Paramecium histone* chaperone Spt16-1 is required for Pgm endonuclease function in programmed genome rearrangements. PLoS Genet..

[101] Matsuda A., Forney J.D (2006). The SUMO pathway is developmentally regulated and required for programmed DNA elimination in *Paramecium tetraurelia*. Eukaryotic Cell.

[102] Ninova M., Holmes H., Lomenick B., Fejes Tóth K., Aravin A.A (2023). Pervasive SUMOylation of heterochromatin and piRNA pathway proteins. Cell Genom..

[103] Andreev V.I., Yu C., Wang J., Schnabl J., Tirian L., Gehre M. (2022). Panoramix SUMOylation on chromatin connects the piRNA pathway to the cellular heterochromatin machinery. Nat. Struct. Mol. Biol..

[104] Tabara H., Mitani S., Mochizuki M., Kohara Y., Nagata K (2023). A small RNA system ensures accurate homologous pairing and unpaired silencing of meiotic chromosomes. EMBO J..

[105] Ward J.R., Khan A., Torres S., Crawford B., Nock S., Frisbie T. (2022). Condensin I and condensin II proteins form a LINE-1 dependent super condensin complex and cooperate to repress LINE-1. Nucleic Acids Res..

[106] Loubalova Z., Konstantinidou P., Haase A.D (2023). Themes and variations on piRNA-guided transposon control. Mob. DNA.

[107] Allen S.E., Hug I., Pabian S., Rzeszutek I., Hoehener C., Nowacki M (2017). Circular concatemers of ultra-short DNA segments produce regulatory RNAs. Cell.

[108] Stefanov B.A., Ajuh E., Allen S., Nowacki M (2024). Eukaryotic release factor 1 from Euplotes promotes frameshifting at premature stop codons in human cells. iScience.

[109] Swart E.C., Serra V., Petroni G., Nowacki M (2016). Genetic codes with no dedicated stop codon: context-dependent translation termination. Cell.

[110] Stefanov B.A., Fussenegger M (2022). Biomarker-driven feedback control of synthetic biology systems for next-generation personalized medicine. Front. Bioeng. Biotechnol..

[111] Stefanov B.A., Mansouri M., Charpin-El Hamri G., Fussenegger M (2022). Sunlight-controllable biopharmaceutical production for remote emergency supply of directly injectable therapeutic proteins. Small.

[112] Stefanov B.A., Teixeira A.P., Mansouri M., Bertschi A., Krawczyk K., Hamri G.C.E. (2021). Genetically encoded protein thermometer enables precise electrothermal control of transgene expression. Adv. Sci..

[113] Huang S., Tao X., Yuan S., Zhang Y., Li P., Beilinson H.A. (2016). Discovery of an active RAG transposon illuminates the origins of V(D)J recombination. Cell.

[114] Wiegand T., Hoffmann F.T., Walker M.W.G., Tang S., Richard E., Le H.C. (2023). Emergence of RNA-guided transcription factors via domestication of transposon-encoded TnpB nucleases. bioRxiv.

[115] Yoon P.H., Skopintsev P., Shi H., Chen L., Adler B.A., Al-Shimary M. (2023). Eukaryotic RNA-guided endonucleases evolved from a unique clade of bacterial enzymes. Nucleic Acids Res..

[116] Jiang K., Lim J., Sgrizzi S., Trinh M., Kayabolen A., Yutin N. (2023). Programmable RNA-guided DNA endonucleases are widespread in eukaryotes and their viruses. Sci. Adv..

[117] Singh M., Seah B.K.B., Emmerich C., Singh A., Woehle C., Huettel B. (2023). Origins of genome-editing excisases as illuminated by the somatic genome of the ciliate *Blepharisma*. Proc. Natl. Acad. Sci. U.S.A..

[118] Seah B.K.B., Swart E.C (2023). When cleaning facilitates cluttering - genome editing in ciliates. Trends Genet..

[119] Kohri N., Ota M., Kousaku H., Minakawa E.N., Seki K., Tomioka I (2023). Optimization of piggyBac transposon-mediated gene transfer method in common marmoset embryos. Plos One.

[120] Shen D., Song C., Miskey C., Chan S., Guan Z., Sang Y. (2021). A native, highly active Tc1/mariner transposon from zebrafish (ZB) offers an efficient genetic manipulation tool for vertebrates. Nucleic Acids Res..

[121] Li M.A., Pettitt S.J., Eckert S., Ning Z., Rice S., Cadiñanos J. (2013). The piggyBac transposon displays local and distant reintegration preferences and can cause mutations at noncanonical integration sites. Mol. Cell. Biol..

[122] Lampe G.D., King R.T., Halpin-Healy T.S., Klompe S.E., Hogan M.I., Vo P.L.H. (2024). Targeted DNA integration in human cells without double-strand breaks using CRISPR-associated transposases. Nat. Biotechnol..

[123] Tou C.J., Orr B., Kleinstiver B.P (2023). Precise cut-and-paste DNA insertion using engineered type V-K CRISPR-associated transposases. Nat. Biotechnol..

[124] Hsieh S.C., Peters J.E (2024). Natural and engineered guide RNA-directed transposition with CRISPR-associated Tn7-like transposons. Annu. Rev. Biochem..

[125] Ran F.A., Hsu P.D., Lin C.Y., Gootenberg J.S., Konermann S., Trevino A.E. (2013). Double nicking by RNA-guided CRISPR Cas9 for enhanced genome editing specificity. Cell.

[126] Arévalo S., Pérez Rico D., Abarca D., Dijkhuizen L.W., Sarasa-Buisan C., Lindblad P. (2024). Genome engineering by RNA-guided transposition for *Anabaena* sp. PCC 7120. ACS Synth. Biol..

[127] Vo P.L.H., Acree C., Smith M.L., Sternberg S.H (2021). Unbiased profiling of CRISPR RNA-guided transposition products by long-read sequencing. Mob. DNA.

[128] Newton M.D., Losito M., Smith Q.M., Parnandi N., Taylor B.J., Akcakaya P. (2023). Negative DNA supercoiling induces genome-wide Cas9 off-target activity. Mol. Cell.

